# Supplementary material for: Live calcium and mitochondrial imaging in the enteric nervous system of Parkinson patients and controls
Source: eLife. 2017 Aug 21;6:e26850. doi: 10.7554/eLife.26850 (PMC5565316; doi:10.7554/eLife.26850)
Supplement: Supplementary file 1. — Clinical data of individual PD patients. SCOPA total, *Patient six entered ‘not applicable’ for the 2 SCOPA items related to sexual function. Mean ± SD are shown. For Hoehn-Yahr scores, median and interquartile range are shown instead of average and SD. DOI: http://dx.doi.org/10.7554/eLife.26850.023 [file elife-26850-supp1.docx]

|  | Gender | Age at onset | Disease duration (years) | UPDRS III (off) | HY (off) | SCOPA total | SCOPA GI | LED (mg) | MMSE |
| --- | --- | --- | --- | --- | --- | --- | --- | --- | --- |
| 1 | M | 59 | 12 | 22 | 2 | 17 | 2 | 947 | 30 |
| 2 | F | 61 | 9 | 14 | 2 | 15 | 4 | 510 | 30 |
| 3 | M | 44 | 17 | 46 | 4 | 11 | 2 | 1740 | 30 |
| 4 | M | 45 | 3 | 12 | 2 | 8 | 0 | 310 | 28 |
| 5 | F | 43 | 6 | 14 | 2 | 11 | 4 | 465 | 30 |
| 6 | M | 69 | 2 | 27 | 3 | 6 * | 4 | 510 | 30 |
| 7 | M | 61 | 9 | 37 | 3 | 18 | 2 | 915 | 30 |
| 8 | M | 41 | 9 | 15 | 2 | 28 | 8 | 610 | 30 |
| 9 | M | 36 | 9 | 24 | 2 | 7 | 1 | 565 | 30 |
| 10 | M | 44 | 11 | 36 | 2 | 32 | 4 | 1215 | 30 |
| 11 | M | 54 | 2 | 23 | 2 | 4 | 1 | 205 | 29 |
| 12 | M | 49 | 7 | 17 | 2 | 2 | 1 | 510 | 27 |
| 13 | F | 61 | 8 | 26 | 3 | 13 | 6 | 445 | 30 |
| 14 | M | 52 | 7 | 23 | 3 | 2 | 1 | 600 | 30 |
| 15 | F | 48 | 6 | 13 | 2 | 4 | 0 | 715 | 30 |
| average | | 51.1 | 7.8 | 23.3 | 2 | 12.3 | 2.7 | 684.1 | 29.6 |
| STDEV | | 9.4 | 3.9 | 10.0 | IQR:2-3 | 9.2 | 2.3 | 388.5 | 0.9 |

**Supplementary file1 | Clinical characteristics of individual PD patients.**

Clinical data of individual PD patients. SCOPA total, *Patient 6 entered ‘not applicable’ for the 2 SCOPA items related to sexual function. Mean ± SD are shown. For Hoehn-Yahr scores, median and interquartile range are shown instead of average and SD.
