# Supplementary material for: Live calcium and mitochondrial imaging in the enteric nervous system of Parkinson patients and controls
Source: eLife. 2017 Aug 21;6:e26850. doi: 10.7554/eLife.26850 (PMC5565316; doi:10.7554/eLife.26850)
Supplement: Supplementary file 2. — Spearman R-values of correlations between Ca2+ imaging parameters and clinical characteristics of PD patients (gray shaded rows) and where applicable (age, SCOPA) of controls (white rows). DOI: http://dx.doi.org/10.7554/eLife.26850.024 [file elife-26850-supp2.docx]

| Correlations calcium imaging | | Age | Disease duration | UPDRS III  (off) | HY (off) | SCOPA total | SCOPA GI | LED (mg) | MMSE |
| --- | --- | --- | --- | --- | --- | --- | --- | --- | --- |
| High K + | % responding neurons | *0.12* | *-0.18* | *0.25* | *0.31* | *-0.17* | *-0.06* | *0.01* | *-0.04* |
|  |  | *-0.14* |  |  |  | *0.57* | *0.06* |  |  |
|  | [Ca^2+^]_i_ transient amplitude | *-0.32* | *-0.11* | *0.03* | *0.08* | *-0.27* | *-0.54* | *-0.03* | *-0.43* |
|  |  | *0.21* |  |  |  | *-0.35* | *0.39* |  |  |
| DMPP | % responding neurons | *-0.08* | *-0.39* | *0.05* | *-0.20* | *-0.12* | *-0.41* | *-0.18* | *0.08* |
|  |  | *0.02* |  |  |  | *0.45* | *0.11* |  |  |
|  | [Ca^2+^]_i_ transient amplitude | *-0.11* | *0.27* | *0.09* | *-0.19* | *-0.39* | *-0.06* | *0.31* | *-0.26* |
|  |  | *0.02* |  |  |  | *0.03* | *0.28* |  |  |
| Electrical stimulation | % responding neurons | *-0.27* | *0.02* | *-0.73* | *-0.78* | *-0.44* | *-0.18* | *-0.22* | *-0.33* |
|  |  | *0.23* |  |  |  | *0* | *0.41* |  |  |
|  | [Ca^2+^]_i_ transient amplitude | *-0.40* | *0.52* | *0.93* | *-0.10* | *-0.21* | *-0.18* | *0.39* | *-0.74* |
|  |  | *0.04* |  |  |  | *-0.01* | *0.28* |  |  |

Spearman R-values of correlations between Ca^2+^ imaging parameters and clinical characteristics of PD patients (gray shaded rows) and where applicable (age, SCOPA) of controls (white rows).
