# Supplementary material for: Live calcium and mitochondrial imaging in the enteric nervous system of Parkinson patients and controls
Source: eLife. 2017 Aug 21;6:e26850. doi: 10.7554/eLife.26850 (PMC5565316; doi:10.7554/eLife.26850)
Supplement: Supplementary file 3. — Spearman R-values of correlations between mitochondrial imaging parameters and clinical characteristics of PD patients (gray shaded rows) and where applicable (age, SCOPA) of controls (white rows). DOI: http://dx.doi.org/10.7554/eLife.26850.025 [file elife-26850-supp3.docx]

| Correlations  mitochondrial imaging | Age | Disease duration | UPDRS III  (off) | HY (off) | SCOPA total | SCOPA  GI | LED  (mg) | MMSE |
| --- | --- | --- | --- | --- | --- | --- | --- | --- |
| mitochondrial volume ratio | *0.23* | *-0.06* | *0.40* | *0.06* | *-0.16* | *0.09* | *0.33* | *0.14* |
|  | *0.37* |  |  |  | *-0.02* | *-0.12* |  |  |
| Mitochondrial density | *0.15* | *0.01* | *0.47* | *0.22* | *-0.42* | *-0.26* | *0.35* | *-0.41* |
|  | *-0.14* |  |  |  | *-0.29* | *-0.19* |  |  |
| Individual mitochondrial volume | *0.22* | *-0.08* | *0.32* | *0.03* | *0.01* | *0.22* | *0.16* | *0.41* |
|  | *0.70* |  |  |  | *0.11* | *-0.16* |  |  |
| TMRE intensity | *-0.45* | *-0.16* | *-0.42* | *-0.32* | *0.52* | *0.50* | *-0.54* | *0.68* |
|  | *-0.49* |  |  |  | *-0.21* | *0.30* |  |  |
| TMRE fluctuations | *-0.43* | *-0.33* | *-0.64* | *-0.38* | *0.41* | *0.26* | *-0.34* | *0.70* |
|  | *-0.56* |  |  |  | *-0.42* | *0.18* |  |  |

**Supplementary file 3 | Correlations between mitochondrial imaging data and PD characteristics**

Spearman R-values of correlations between mitochondrial imaging parameters and clinical characteristics of PD patients (gray shaded rows) and where applicable (age, SCOPA) of controls (white rows.
