# Supplementary material for: Live calcium and mitochondrial imaging in the enteric nervous system of Parkinson patients and controls
Source: eLife. 2017 Aug 21;6:e26850. doi: 10.7554/eLife.26850 (PMC5565316; doi:10.7554/eLife.26850)
Supplement: Supplementary file 4. — Spearman R-values of immunofluorescent counting correlated with clinical characteristics of the PD patients (gray shaded rows) and where applicable (age, SCOPA) of controls (white rows). DOI: http://dx.doi.org/10.7554/eLife.26850.026 [file elife-26850-supp4.docx]

| Correlations immunohistochemistry | Age | Disease duration | UPDRS III  (off) | HY (off) | SCOPA total | SCOPA GI | LED (mg) | MMSE |
| --- | --- | --- | --- | --- | --- | --- | --- | --- |
| Number of neurons / ganglion | *-0.10* | *0.14* | *-0.11* | *-0.42* | *0.05* | *-0.32* | *-0.14* | *-0.34* |
|  | *-0.16* |  |  |  | *-0.13* | *0.41* |  |  |
| Total number of neurons | *-0.09* | *0.05* | *-0.36* | *-0.72* | *-0.08* | *-0.25* | *-0.13* | *-0.35* |
|  | *-0.09* |  |  |  | *0.02* | *0.40* |  |  |
| Individual neurons | *0.26* | *0.21* | *-0.16* | *-0.44* | *0.07* | *-0.15* | *0.07* | *-0.47* |
|  | *-0.04* |  |  |  | *-0.12* | *0.25* |  |  |
| Number of ganglia | *-0.02* | *0.06* | *-0.31* | *-0.64* | *-0.09* | *-0.10* | *-0.04* | *-0.39* |
|  | *-0.02* |  |  |  | *-0.05* | *0.38* |  |  |

**Supplementary file 4 | Correlations between immunohistochemical data and PD characteristics**

Spearman R-values of immunofluorescent counting correlated with clinical characteristics of the PD patients (gray shaded rows) and where applicable (age, SCOPA) of controls (white rows).
